# Comparison of dental effects of traditional and clear twin block: A randomized clinical trial

**DOI:** 10.34172/joddd.025.41445

**Published:** 2025-03-31

**Authors:** Ahmad Behroozian, Anahita Pourahmad, Mahsa Zali, Amir Zandesh

**Affiliations:** ^1^Department of Orthodontics, Faculty of Dentistry, Tabriz University of Medical Sciences, Tabriz, Iran; ^2^Dental Student, Faculty of Dentistry, Tabriz University of Medical Sciences, Tabriz, Iran; ^3^Department of Speech Therapy, School of Rehabilitation, Tehran University of Medical Sciences, Tehran, Iran

**Keywords:** Clear twin block, Dental, Skeletal Cl II, Traditional twin block

## Abstract

**Background.:**

Growth modification is a strategy for the treatment of skeletal Cl II patients. Clear twin block (CTB) is a modification of traditional twin block (TTB), which is made from thermoplastic sheets instead of acrylic resin and wire. This study compared the dental effects of the clear and TTBs.

**Methods.:**

In this randomized clinical trial, 60 growing skeletal Cl II patients with dental Cl II div 1 malocclusion were selected and randomly divided into two groups: TTB and CTB. Cephalometric radiography and stone models were taken before treatment (T_1_) and six months (T_2_) after appliance insertion. Data were analyzed using independent t-test and paired t-test at the 0.05 significance level.

**Results.:**

There was a significant difference between CTB and TTB in IMPA at T_2_ (*P*<0.05). Intragroup comparisons showed no significant change from T_1_ to T_2_ in the CTB group and for U1-SN and U1-Pal amounts in the TTB group. T1-T2 comparison showed a significant change in IMPA in the TTB group (*P*<0.05).

**Conclusion.:**

TTB showed more protrusion of lower incisors compared to CTB.

## Introduction

 The skeletal Cl II phenotype is characterized by a combination of maxillary and mandibular retrognathism. Skeletal Cl II malocclusions account for over one-third of all malocclusions.^1^

 It is one of the common skeletal problems in orthodontic patients and can be seen in either skeletal or dental form, each presenting with unique clinical manifestations.^2^ The short-term evidence indicates that growth modification with removable functional appliances is efficacious in improving skeletal Cl II patients; however, their effects are mainly dentoalveolar rather than skeletal.^3^

 Growth modification involves modifying the amount and direction of mandibular growth by utilizing functional appliances.^4,5^ Various functional appliances have been developed to treat skeletal Cl II problems. The twin block, introduced by Clark, is one of the widely used functional appliances in treating these patients.^6^ Several studies have shown the beneficial effects of the twin block in the treatment of skeletal Cl II patients.^7,8^ However, wire components of the appliance, like clasps and labial bow, can irritate tissues and may require frequent adjustments. Furthermore, the twin block’s wire components and bulky structure may interfere with speech and esthetics. This, in turn, may decrease patient cooperation and treatment efficiency.^9,10^ Furthermore, there is general agreement that these appliances cause proclination of mandibular incisors and retroinclination of maxillary incisors.^3^

 Clear aligners and retainers have been met with great success, and the clinicians have introduced several modified appliances made from thermoplastic sheets instead of wires and acrylics. The clear twin block (CTB), introduced by Behroozian et al, is made from thermoplastic clear sheets and has the exact mechanism as the traditional twin block (TTB). The theory behind CTB is to improve the appearance of the TTB and increase patient cooperation.^9^

 Protrusion of lower incisors and retrusion of upper incisors are mentioned as dental side effects of functional appliances.^11^ However, few clinical trials have compared the dental effects of the traditional and CTB appliances.

## Methods

 Sixty patients with skeletal Cl II div 1 problem were selected and randomly divided into CTB and TTB groups in this randomized clinical trial. Written informed consent was obtained from parents or legal guardians before enrolling the participants in the study. The patents were randomly allocated to CTB and TTB groups. Growing patients were selected according to cervical vertebral maturation (stages II and III). The presence and full eruption of the first permanent molars and upper and lower incisors were necessary for the fabrication of the appliance and data acquisition.

 Patients who did not adhere to regular visits and full-time wear were excluded. Any breakage of the appliance, like breakage of the clasps or acrylic portion, and losing the appliance led to exclusion from the study.

**Figure 1:**
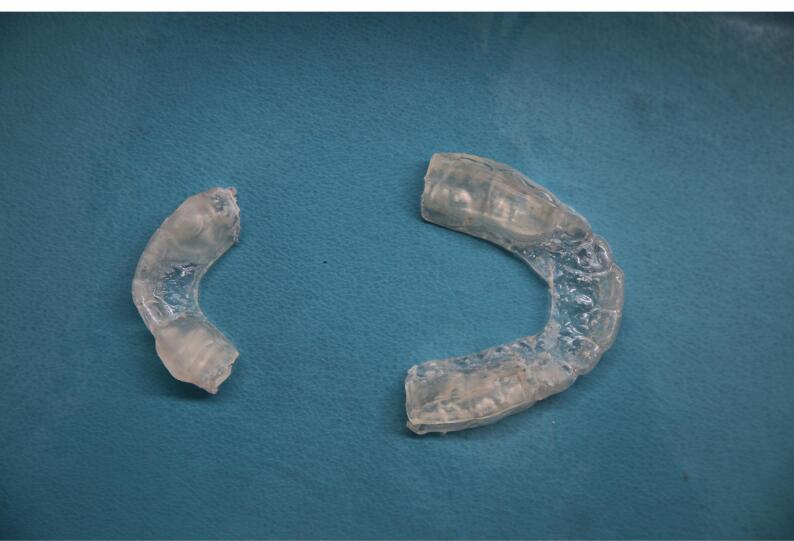
The clear twin block is made from thermoplastic sheets instead of metal wires and acrylic resin

 After taking the impression with alginate, the impression was sent to a dental laboratory. CTBs were fabricated based on the method presented by Behroozian and Kalman.^9^ Health education and methods of use were provided for all patients at the appliance delivery session. Instructions were repeated at follow-up sessions (Figure 1).

 Data were collected from the patients at the first session (T_1_) and six months after delivering the appliances (T_2_). Data were analyzed using independent t-test and paired t-test at the 0.05 significance level.

## Results

 Sixty (30 CTB and 30 TTB) subjects completed the study and were analyzed for dental side effects. The mean cephalometric findings are shown in Tables 1 and 2.

**Table 1:** Comparison of the amounts of IMPA, U1SN and U1-PAL in different observation periods

**Appliance**		**T1**		**T2**		***P*** ** value**
		**Mean**	**SD**	**Mean**	**SD**	
CTB	IMPA	92.0167	6.27746	92.8333	5.06957	0.41
	U1SN	113.9667	4.00832	114.1333	3.99350	0.81
	U1PAL	61.6000	2.59203	61.4167	2.52641	0.71
TTB	IMPA	92.7333	4.56541	96.3167	4.31588	0.00*
	U1SN	113.8167	4.16398	113.6333	3.95297	0.79
	U1PAL	61.3167	2.61347	61.2833	2.40121	0.93

*P <0.05 was considered significant; CTB: clear twin block; TTB: traditional twin block.

**Table 2:** Comparison of the amounts of IMPA, U1SN and U1-PAL in clear twin block and traditional twin block

**Time point**		**CTB**		**TTB**		***P*** ** value**
		**Mean**	**SD**	**Mean**	**SD**	
T1	IMPA	92.0167	6.27746	92.7333	4.56541	0.47
	U1SN	113.9667	4.00832	113.8167	4.16398	0.84
	U1PAL	61.6000	2.59203	61.3167	2.61347	0.55
T2	IMPA	92.8333	5.06957	96.3167	4.31588	0.00*
	U1SN	114.1333	4.00832	113.8167	3.95297	0.49
	U1PAL	61.6000	2.59203	61.3167	2.61347	0.76

*P <0.05 was considered significant; CTB: clear twin block; TTB: traditional twin block.

 There was no significant difference between the dental criteria of the two groups at T_1_ (*P* > 0.05). The authors used paired t-test to compare dental criteria between T_1_ and T2. T_1_-T_2_ comparison showed a significant change in IMPA in the TTB group (*P* < 0.05). The intra-group comparisons showed no significant change from T_1_ to T_2_ in the CTB group. Independent t-test was used to compare inert-group differences. There was a significant difference between the CTB and TTB groups in IMPA amounts in T_2_ (*P* < 0.05). U1-SN and U1-Pal showed no significant difference in inter-group comparison at T_2_.

## Discussion

 Growth modification is the method of choice for skeletal Cl II malocclusion patients in circumpubertal ages, and twin block is one of the most commonly used functional appliances for this purpose.^5^ Many studies have reported that the twin block appliance is effective in managing skeletal Cl II patients.^7,8^

 CTB is a modification of TTB, which is made from clear thermoplastic sheets instead of acrylic plates and wires.^9^ Behroozian et al^12^ showed that CTB and TTB can change the muscular activity of circumoral muscles. They concluded that this ability indicates that CTB can be as successful as TTB in treating skeletal Cl II patients. Patient cooperation is a crucial factor in the success rate of removable appliances.^4^ Speech problems are a deterrent factor that can reduce patient cooperation.^13^ This, in turn, may inhibit full-time wear of the appliance, especially during school hours or social communications.Therefore, one of the critical features in designing removable appliances, especially functional appliances, is the effect of that appliance on speech.

 Because of its novelty, some questions about the efficacy of CTB need to be surveyed. One of these questions is comparing the dental effects of CTB and TTB in treating skeletal Cl II patients. In this randomized clinical trial, we found that the protrusion of the lower incisors in patients using TTB was significantly greater than in those using CTB, which can be attributed to the full crown converge of thermoplastic sheets in CTB. Traditionally, one of the routine methods to decrease lower incisors’ proclamation is to use a Clarck twin block to increase the coverage of the crown of the lower anterior teeth.^14^ In functional appliances, the soft tissue stretch exerts a forward force on the lower incisors. In TTBs, the labial bow has a single contact point on the buccal surface of the anterior teeth. In contrast, the thermoplastic sheet of CTB has a two-point contact with the buccal surfaces of the crowns; therefore, the forward bodily movement of the teeth will happen with CTB use. On the other hand, more tipping of anterior teeth is anticipated with TTB use. The increase in IMPA showed the tipping of lower incisors with TTB use.

 Why are the protrusion of lower incisors and retrusion of the lower incisors side effects of functional appliances? Weschler and Pancherz et al^15^ showed that the dental side effects of functional appliances decreased the potential for skeletal changes and growth modification. It means that overjet correction in patients is a combined effect of dental tipping and skeletal growth during growth modification. Since the amount of overjet correction is a pre-determined value for each patient, the potential for skeletal change decreases with increased dental tipping.

 It should be remembered that sometimes, the protrusion of lower incisors and retrusion of upper incisors help treat the patients. In skeletal Cl II patients with protruded and flared upper incisors and retruded lower incisors, a functional appliance that could correct skeletal Cl II and simultaneously retract upper incisors and protrude lower incisors is a double blessing. The expert clinician can do this by trimming the acrylic resin from the lingula region of the upper incisors and adjusting the labial bow. These mechanics are not feasible in CTB. Therefore, although dental side effects reduce the potential for growth modification and skeletal changes, they can be advantageous in selected cases.

 El Kattan et al^16^ introduced a method for fabricating a modified twin block. The study’s control group was a no-treatment group, and the modified twin block patients were not compared with TTB patients.In addition, the method of fabrication of the CTB was fundamentally different from the method presented by Behroozian and Kalman.^9^ El Kattan et el mounted the stone models in the articulator using a construction bite. Then, they formed bite planes with a self-cured acrylic resin. The sheets were then formed over the casts and bite plates. The authors believe that forming the sheet on the bite planes disrupts the accuracy of the construction bite because the sheets have their own thickness, and this added material will disturb the pre-determined position of the jaws. In the present study, the sheets were formed over the stone models, and then the acrylic resin bite plates were added to the sheets in the articulated position so the articulation relationship was not disturbed.^9^ In the method suggested by Golfeshan et al,^17^ the bite was registered while the clear sheets were put on the upper and lower arches in the patient’s mouth, and two sessions were needed for bite registration. However, in the present study, impression-taking and bite registration were done in one session. In contrast to Golfeshan and colleagues’ study, in the method introduced by Behroozian and Kalman, the undercuts were not blocked out with molten; therefore, the retention of the appliance was not affected. Golfeshan et al took the second cephalometry after treatment (when the overjet decreased to 0‒1 mm). Since all the patients cannot finish the treatment at the same time, the treatment duration will be different in different patients, which, in turn, can influence dental side effects. Therefore, in the present study, we decided to settle the T_2_ six months after treatment, which was equal for all of the patients, and the confounding variable of time was eliminated.

## Limitations and Suggestions

1. The authors suggest a more sensitive case selection, especially regarding age, sex, and cervical vertebral maturation stage.
2. The patients received detailed education about the method and duration of the wearing of the appliance. However, the authors had no method to control and measure the wear time of the patient. Since wear time is critical to the success of removable appliances, we suggest excluding patients with poor cooperation from the study.

## Conclusion

 The patients using CTB showed less tipping of lower anterior teeth compared to the TTB group. This does not mean the CTB is necessarily better for all patients. The clinician can choose between TTB and CTB based on whether tipping of anterior teeth is needed.

## Competing Interests

 The authors declare no competing interests.

## Ethical Approval

 This study was approved by the Ethics Committee of Tabriz University of Medical Sciences (IR.TBZMED.REC.1400.730). The approval code for RCT registration is IRCT20170723035238N3.
